# Biogenic nanoparticles as a promising drug delivery system

**DOI:** 10.1016/j.toxrep.2024.101887

**Published:** 2024-12-31

**Authors:** Rehab M. Abdel-Megeed

**Affiliations:** Therapeutic Chemistry Department, National Research Center, El Buhouth St., Dokki, Cairo 12622, Egypt

**Keywords:** Biogenic nanoparticles, Drug Delivery system, Bionanotechnology

## Abstract

Nanotechnology has significantly influenced the worldwide medical services sector during the past few decades. Biological collection approaches for nanoparticles are economical, non-toxic, and ecologically benign. This review provides up-to-date information on nanoparticle production processes and biological sources, including algae, plants, bacteria, fungus, actinomycetes, and yeast. The biological technique of generating nanoparticles has advantages over chemical, physical, and biological methods, including low-toxicity and friendly to the environment, thereby providing a viable option for therapeutic applications as s promising drug delivery system. In addition to aiding researchers, the bio-mediated, obtained nanoparticles also modify particles to promote both health and safety. We also looked at the important medicinal uses of nanoparticles, including their antifungal, antimicrobial, antiviral, antidiabetic, anti-inflammatory, and antioxidant properties. The current study highlights the findings of recent research in this field and discusses various methods proposed to describe the bio-mediated acquisition of novel nanoparticles.. The production of nanoparticles via biogenic sources possess various benefits, such as low cost, bioavailability, and environmental friendliness. In addition to the determination of the bioactive chemicals mediated by nanoparticle as well as the examination of the biochemical pathways and enzyme reactions. The major focus of this review is highlighting on the essential role of biogenic nanoparticles as promising drug delivery system.

## Highlights

• Biological collection of nanoparticles are economical, non-toxic, and ecologically benign.
• Highlights on synthesis, characterization, types and applications of biogenic nanomaterials.
• Clarifying antifungal, antimicrobial, antiviral, and antioxidant potential of biogenic nanoparticles.
• Elucidate anticancer potential and antidiabetic effect of biologically produced nanoparticles.
• Role of Egyptian research in the field.

## Graphical Abstract

**Figure:**
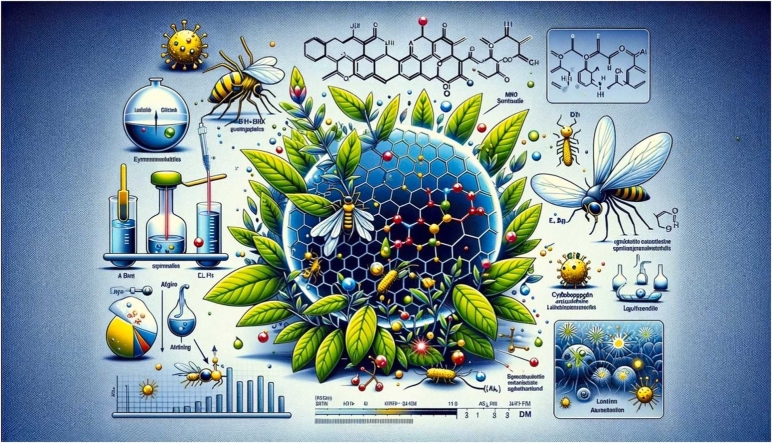

## 1 Introduction to bionanotechnology

The term "nano" originates from the Greek word "nanos", which meaning “little”. Their particles having at least one dimension ranging from 1 to 100 nm [1]. Nonetheless, the bulk of materials used in drug delivery fall between the 10–200 nm range. These particles can modify their chemical and physical properties due to their distinct electronic structure, huge conductivity, wide outside position, and quantum size importance. Nowadays, nanoparticles are applied in many fields, such as fabrics, electronics, skincare products, and anti-viral [2], [3]. Nanotechnology is a phrase used to designate areas of science and engineering where phenomena occurring at nanoscale dimensions are exploited in the design, characterization, manufacturing, and applications of materials, structures, devices, and systems. Nanotechnology have several positive impacts by researchers, and it is currently widely used. The remarkable features of the small size of nanoparticles are crucial in a variety of reactions. The findings of nanotechnology and nanomedicines are so extensive and diverse. Impressive advancements in nanomedicine have elevated the medication to a new level with noteworthy medical results. Research on the substantial potential of nanotechnology in healthcare is necessary. Numerous studies are being conducted in the medical field to investigate optimal techniques and approaches, such as cancer treatment, cardiovascular disease therapeutic gene, and nephrology. Traditional treatment has advanced significantly, and nanotechnology and nanoparticle quality have both improved and produced promising outcomes. Nanomedicines have also been used in gene therapy. Numerous studies concentrated on the use of viral vectors thought to be drug delivery systems [4]. Scientists are becoming more interested in nanoparticles, which are nanoscale platforms with a wide range of potential sizes and configurations for sophisticated applications in biotechnology. The unique thermodynamic and radiative features of nanoparticles may make them useful in applications like diagnosis and pharmaceuticals. Compared to single molecules or bulk particles, these characteristics give nanoparticles more focused and profound effects. It is particularly useful in the fields of medical, textiles, safeguarding, food production, agriculture, skincare products, and exploration of space because of its enhanced electric transport, stimulated texture, and stability [5]. When compared to other bulk materials, nanoparticles have amazing shape and size properties, a wide range of applications, and are utilized for extensive applications [6].

Compared to single molecules or bulk particles, these characteristics give nanoparticles more focused and profound effects. It is particularly useful in the fields of medical, textiles, safeguarding, food production, agriculture, skincare products, and exploration of space because of its enhanced electric transport, stimulated texture, and stability. When compared to other bulk materials, nanoparticles have amazing shape and size properties, a wide range of applications, and are utilized for extensive applications [6].

Large-scale approaches to biology and nanotechnology divided into two categories: first, using tools and techniques inspired by nanomaterials in biological systems; and second, using biological systems as models for the development of nanoproducts. High stability, adaptability, and target selectivity are just a few of the benefits that nanomaterials provide over conventional biotechnological techniques. Because of its numerous applications across a wide variety of majors, nanotechnology can support the global economy and a sustainable future [7].

Nanomaterials have several applications in medicine and healthcare, including drug delivery, therapeutic, and diagnostic activities.

Prior to determining the patient's condition, medical experts must diagnose diseases based on the presence of visual symptoms. However, treatment might not be as successful as it could have been by the time those symptoms manifest.

However, conventional detection methods are mostly depending on the normal organic molecules, which reduce their specificity as it breaks down more quickly; nanomaterials can resist a significantly higher number of cycles of excitations and light emissions. Furthermore, diagnostic nanotechnology based on gold nanoparticles declared to be high sensitive as a target specific probe. It is also beneficial in Cancer detection as well as viral infection.

## 2 Therapeutic applications of nanomaterials and drug delivery

Therapeutic delivery of nanoparticles enables the delivery of medications to specific locations that are frequently challenging conventional medications to reach. As radio or magnetic signals can direct the therapeutic dose to the disease location if it is loaded as a nanoparticle. Furthermore, nano-drugs evaluated to release exclusively in response to certain molecules or outside stimuli. Furthermore, it is possible to lower effective dosages in order to prevent negative side effects from strong medications. By encasing medications in nanoparticles and delivering them to the intended location, it is now able to regulate drug release with far greater precision than in the past. Drug delivery system based on nanotechnology can ameliorate bioavailability of various therapeutic agents that limited in orally administration due to their poor bioavailability. Numerous studies and optimization attempts are being carried out to fine-tune the size, content, and surface charge of these nanoparticles in order to guarantee their efficacy and safety. To guarantee their safety in clinical applications, researchers are also looking into their possible toxicity and biocompatibility [2]. to create drug delivery systems that minimize side effects, maximize treatment effectiveness, and efficiently transport therapeutic molecules to the intended spot. Understanding biological interactions and improving the design of lipid-based nanoparticles need interdisciplinary cooperation between chemists, biologists, and doctors [3]. While biologists investigate intracellular trafficking and cellular uptake, chemists can create and synthesize different lipid compositions to improve stability and biocompatibility. In preclinical models or clinical trials, clinicians can assess therapeutic efficacy and potential toxicity, offering insightful input for additional nanoparticle design optimization. Nano-formulations not only shield substances that are vulnerable to degradation in high pH conditions, but they can also prolong the half-life of medications by enhancing their bio-adhesion and retention in the body.

**Fig. 1:**
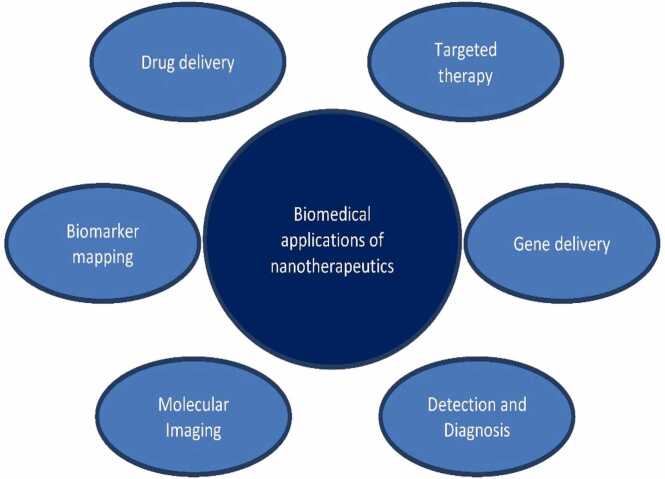
General application of nanotechnology [13].

When used as drug delivery vehicles, nanoparticles can improve a number of critical characteristics of free medicines, including solubility, pharmacokinetics, biodistribution, and in vivo stability [8]. Because of these benefits, nanoparticles was employed as possible drug delivery vehicles in this area. A hydrophobic fluorophore was delivered in vitro using mixed monolayer protected gold clusters as an example of cellular delivery [9]. Designing nanoparticles for the expansion of oral medication delivery systems, Pandey and Khuller [10]advised that the development of an acceptable oral dose form for streptomycin and other antibiotics that are, if not exclusively, injectable [10]. Silver nanoparticles interact with HIV-1 in a size-dependent manner, with nanoparticles ranging from one to 10 nm near to the virus. Silver nanoparticles were thought to prevent the HIV-1 virus from attaching to host cells due to their spatial arrangement, center to-center spacing, and bare sulfur-bearing residues on glycoprotein knobs. Currently, most imaging experiments with gold nanoparticles are conducted in cell culture [11]. Modern classes of vaccines are also developed via nanotechnology [12]. The following diagram illustrated the application of nanoparticles in various aspects [Fig. 1]. As illustrated in this figure, targeted drug delivery is a significant biomedical application that seeks to prevent harm to nearby healthy cells while delivering anticancer medications to the precise location of the tumor. Furthermore, Numerous bioimaging methods, including computed tomography (CT), positron emission tomography (PET), magnetic resonance imaging (MRI), and ultrasound, are employed in the diagnosis and detection of illnesses. These methods are non-invasive, and some of them can create interior organ images with excellent resolution. In these bioimaging procedures, contrast chemicals are typically utilized to distinguish between healthy and sick tissue as well as to identify the organ or tissue of interest. It is possible to use nanoparticles to complex viral vectors as well as nucleic acids. Prior to mixing these vectors with the magnetic particles, the gene vector must be prepared as part of the normal process known as magnetic vector complexing. The majority of published findings indicate that extra agents are required to create the complex between the gene delivery vector and the nanoparticles. Polymers have the ability to serve as a connection between the particles and the nucleic acid in DNA.

## 3 Biogenic nanomaterials: synthesis, characterization, and applications

**Fig. 2:**
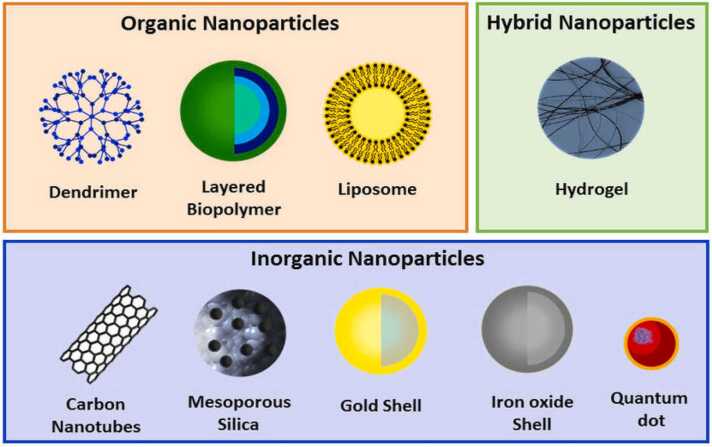
organic and inorganic nanoparticles [30].

Nanoscience studies the properties of materials ranging from 1 to 100 nm, while nanotechnology applies this knowledge to generate or evaluate new products. The ability to alter structures at the level of the atom enables the development of nanomaterials [14]. These materials process distinct optical, magnetic and electrical characters at the nanoscale,and these can be utilized in different fields such as electronics and medicine [15]. Furthermore, scientists divided nanoparticles into Organic and inorganic as described in Fig. 2 [Fig. 2]. Organic nanoparticles represent all the nanomaterials established from carbon compounds, while inorganic nanoparticles represent all the nanomaterials established from non-carbon compounds. One of the benefits of inorganic nanoparticles is that they are non-toxic, hydrophilic, and biocompatible with living systems. When it comes to stability, inorganic nanoparticles are better than organic ones. Furthermore, Inorganic nanoparticles as compared to organic nanoparticles are smaller in size, improve stability, control surface functions and enhance magnetic characteristic features. On the other hand, organic nanoparticles are more biocompatible, biodegradable and nontoxic than inorganic nanoparticles. Inorganic nanoparticles are highly effective for drug delivery among manmade nanomaterials. Inorganic nanoparticles feature distinct material and size properties, a high surface area volume ratio, and variable surfaces that can be customized for drug delivery and targeting. These compounds have targeted and regulated distribution, are easily absorbed, have reduced hazardous potential, improved functional qualities, and are biocompatible. Inorganic nanoparticles are chemically stable and do not deteriorate in the human body's plasma or cytoplasm, ensuring their integrity throughout delivery [16]. Inorganic nanoparticles (NPs) are typically made from noble metals like gold (Au) and silver (Ag). Metals including nickel (Ni), cobalt (Co), iron (Fe), magnetite (Fe3O4), and iron-platinum (FePt) are utilized to create magnetic nanoparticles with superior characteristics in a magnetic field. Fluorescent nanoparticles include quantum dots, silicon dioxide (SiO2), etc [17]. The biocompatibility, hydrophilic nature, shallow toxicity profile, and stability of inorganic nanoparticles have raised concerns about their potential as drug delivery systems and disease regulators because of their resistance to microbial attack. The diameter-tunable characteristics of porous inorganic nanoparticles facilitate the transfer of various tiny medicines to big proteins or oligonucleotide strands. Inorganic nanoparticles' surfaces can be altered to aid in targeted drug delivery and to monitor drug release. Inorganic nanoparticles coupled with drugs offer an adaptable framework for applications for imaging-based therapy and diagnosis. Both inorganic and metallic nanoparticles, such as silicon, silica, and graphene are resistant and stiff. Nevertheless, they are easily mechanically and chemically changed, making them easier to transfer into tumor cells, while their restricted flexibility makes penetration more difficult [18]. Since the free nanoparticles have a distinct surface, they react with biomolecules to produce both radical and non-radical ROS. A benign, biocompatible coating encapsulates the inorganic nanoparticles as a result. By encasing a different metal, core/shell nanoparticles are created, which enhances attributes like photoluminescence and quantum yield. Air stability and fewer surface flaws are provided by the passivation of the non-radiative combination sites. Exocytosis or endocytosis are the two processes that move encapsulated nanoparticles across cells [17]. Green synthesis, or the ecologically benign synthesis of metallic nanoparticles from biological components, has drawn a lot of interest. Green synthesis makes use of biomass, plant extracts, microorganisms, and a variety of other reductants. The term "green nanotechnology" describes the production of nanoparticles from plants and microorganisms like fungi, bacteria, and green algae.

### 3.1 Types of nanoparticles

#### 3.1.1 Metallic nanoparticles

Metallic nanoparticles include silver, zinc, copper, selenium, magnesium, iron, titanium, and gold nanoparticles. It widely used in laser-based therapy as optical biosensors as well as drug delivery tools [19], [20].

### 3.2 Liposomes

Liposomes are spherical, lipid bilayer-containing vesicles with particle sizes ranging from 30 nm to numerous microns. They are characterized by the ability of its surface to be modified with polymers, proteins, or antibodies enhancing macromolecular drugs, including crystalline metals and nucleic acids, to be incorporated into liposomes such as Poly (ethyleneglycol) (such as PEGylated liposomal doxorubicin (Doxil®) [21], [22], [23].

### 3.3 Micelles

They composed of lipids and amphiphilic molecules with a hydrophilic outer layer and a hydrophobic core. Micelles improve bioavailability of drugs. Their diameter ranges from 10 to 100 nm [24], [25].

### 3.4 Carbon nanotubes

They are cylindrical molecules that composed of rolled-up sheets of carbon atoms. Carbon tubes can be used as biological sensors and imaging contrast agents [26], [27].

### 3.5 Green nanotechnology

**Fig. 3:**
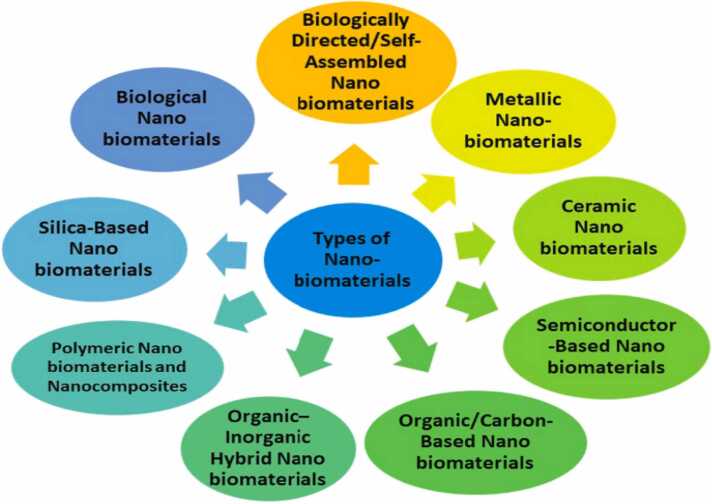
Types of nanoparticles [30].

Green nanotechnology is the application of concepts from green chemistry to nanotechnology. It produces and processes environmentally friendly, safe, affordable, and sustainable nanomaterials [28]. The main target of green nanotechnology is to limit nanomaterials hazard effects [29]. The following figure demonstrated types of nanoparticles [Fig. 3].

### 3.6 Green nanomaterial preparation

Generally, nanomaterials are synthesized by three distinct methods: chemical, biological, and physical. Nanomaterials preparations occurs through two different approaches bottom-up or top-down. Using smaller construction blocks, the bottom-up technique prepares the material. By pulverizing a bulk material into the proper shape and structure, top-down microfabrication techniques have been employed to produce nanomaterial. Green synthesis has several advantages over chemical and physical approaches, including cost-effectiveness, eco-friendliness, easier synthesis at room temperature and pressure, and increased bioactivity and decreased toxicity [31]. In order to transform bulk metal salts into nanoparticles and capping agents that give nanoparticles stability and make them biocompatible for a variety of biological applications, green systems release proteins and enzymes that function as reducing agents.

The widely used green methods for creating nanomaterials are explained as the following: 1- By ionic green solvent 2- Conventional chemical synthesis methods are being replaced by more environmentally friendly techniques such as microwave synthesis, electrochemical synthesis, sonochemical synthesis, solvothermal synthesis, hydrothermal synthesis, and biosynthesis [32]. Biosynthesis is the excellent method for synthesizing green nanomaterials using natural ingredients is a multistep process controlled by catalytic enzymes. Using naturally derived starting materials and low-energy procedures, green synthesis is a technology that is equally as effective as traditional synthesis, if not more so. It offers a sustainable approach to the creation of nanomaterials. It reduces pollution, costs less, and improves environmental and human health safety. A variety of organisms, including bacteria, fungi, actinomycetes, algae, and plants, are used in green biosynthesis to produce nanomaterials. Furthermore, bacteria able to synthesize inorganic compounds both extracellularly and intracellularly. Biosynthesis is commonly used to produce metalic nanoparticles [33]. It has been discovered that Bacillus *sp.* is efficient in producing gold and lead nanoparticles [34]. Gold and silver nanoparticles were also biologically produced by a fungal-mediated green method by *Fusarium oxysporum* and *Aspergillus flavus* [35]. In addition to actinomycetes, the biogenic microorganisms which possess the characteristics of fungi and prokaryotic bacteria. Similarly, algae-based nanomaterial biosynthesis has been accomplished with success [36].

**Fig. 4:**
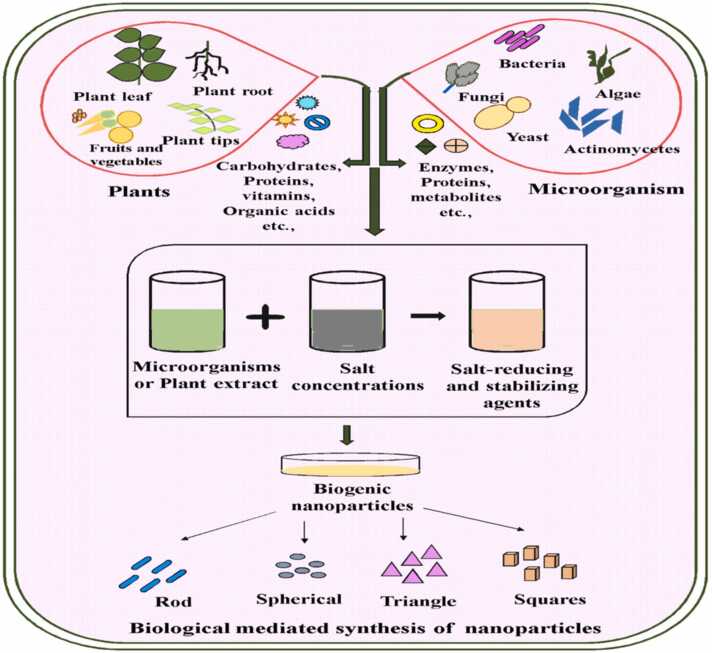
Biological mediated synthesis of nanoparticles [37].

**Fig. 5:**
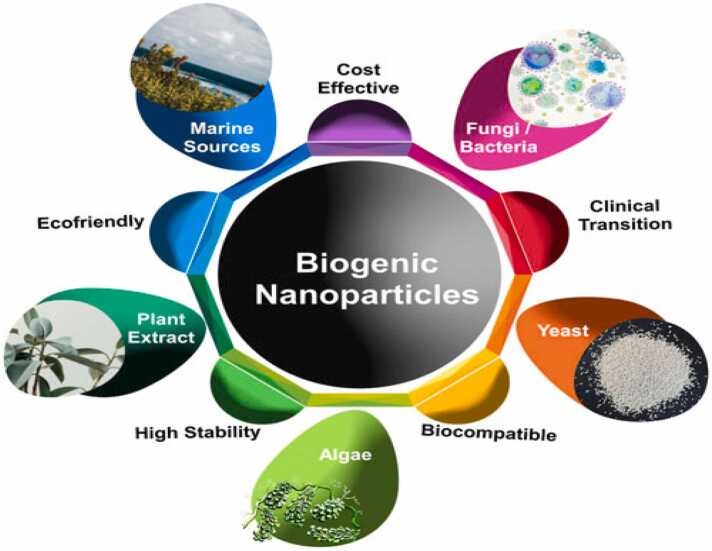
Benefits and sources of biogenic nanoparticles [38].

The following figures represented biogenic method for nanoparticles synthesis and their beneficial features [Fig. 4, Fig. 5].

### 3.7 Fungi-based nanoparticles synthesis

The most prevalent type of microorganisms, fungi are employed in many scientific fields for purposes such as enzyme synthesis, bioremediation, and nanotechnology production [39], [40]. Fungi able to produced nanoparticles that produced by bacterial cells due to their greater capacity to endure metal concentrations and their greater ability to bind with cell walls for metal ions. Mostly filamentous mushrooms, imperfect fungi, and other fungi, ascomycetes produce around 6000 physiologically active chemicals. In addition to being more valuable than bacteria at creating nanoparticles, fungi also have a greater tendency to accumulate metals. During intracellular synthesis, metals were first introduced into the culture and rotated inside the biomass. In order to remove the biomass and release the nanoparticles after biosynthesis, the produced nanoparticles must be extracted using centrifugation, filtration, and chemical processing. Significant advantages include the ease of downstream processing and scaling up, the economic viability, and the presence of mycelia, which offers a greater surface area. Fungal-based NP is produced by a bio-mineralization mechanism that includes multiple metal ion reduction by both extracellular and internal enzymes and biomolecules. Silver was a preferred metal for both research and manufacture of nanoparticles production based on fungi. Then, Ti, Au, Cu, Se and Zn have been utilized by fungus in nanoparticles production [41], [42], [43]. Different fungal species was employed in the biosynthesis of bio nanoparticles such as *Rhizopus, Verticillium, Fusarium, Aspergillus, Penicillium,* and *Trichoderma sp.* [44], [45]. Fungi-derived nanoparticles have been widely applied as an anticancer drug, antivirals, antimicrobials, antifungals, antibiotics, in addition to its commonly application in medicine, disease diagnosis, bio-imaging, agriculture, and industry. Fungal cells generate a significant amount of NPs in comparison to those of bacteria due to the ability of fungi to secrete more proteins leading to greater nanoparticles output [42]. [Table 1].

**Table 1:** Biosynthesis of nanoparticles using fungi.

**Fungi species**	**Type of Nanoparticles**	**Size of Nanoparticles**	**Biological Activities**	**Reference**
*Aspergillus sp.*	Silver, iron	20–40 nm 30–200 nm	Antifungal, Antiviral, Antibacterial, Anticancer	[46], [47], [48], [49]
*Glycosmis mauritiana*	silver	65 nm	antioxidant, antimicrobial, anti-inflammatory, tyrokinase inhibitory activity	[50]
*Penicillium sp.*	Gold	50 nm	Extracellular synthesis	[51], [52]
*Chrysosporium tropicum*	silver	20–50 nm	Drug formation and diseases diagnosis	[53]
*Fusarium oxysporum*	silver	20–50 nm	Drug formation and diseases diagnosis	[52]

### 3.8 Nanoparticles synthesis using yeast

Large-scale extracellular synthesis of nanoparticles with simple post-synthesis handling. Yeast production is simple to control in laboratory settings when producing large quantities of metal nanoparticles, and using basic nutrients and the quick development of yeast strains offer a number of benefits [54], [55].

As represented in Table 2, *Saccharomyces pombe* and *Candida glabrata* yeast have been investigated to produce intracellular synthetized titanium, silver, sulphide, cadmium, gold and selenium nanoparticles [56] (Table 2).

**Table 2:** Yeast-mediated synthesis of nanoparticles.

**Yeast species**	**Type of Nanoparticles**	**Size of Nanoparticles**	**Biological Activities**	**Reference**
*Saccharomyces cerevisiae*	Selenium	30–100 nm	Antimicrobial	[57]
*Saccharomyces cerevisiae*	silver	100 nm	Antibacterial	[58]
*Saccharomyces cerevisiae*	Palladium	10 −100 nm	Photocatalytic activity	[58]

### 3.9 Nanoparticles synthesis using bacteria

Researchers has concerned by synthesizing metallic nanoparticles using bacterial cells [59]. Bacteria are a suitable candidate for nanoparticles synthesis for their widespread presence in the environment and their ability to adapt to challenging conditions. Furthermore, they are affordable to cultivate quick growing, and easy to manage growth conditions including oxygenation, temperature, and incubation period. Different bacterial strains can synthesis inorganic intracellular or extracellular nanoparticles. *Pseudomonas stutzeri* bacteria could produce silver nanoparticles outside the cells. Moreover, various bacterial Gram negative as well as Gram-positive strains can regulate silver nanoparticles biosynthesis such as *B. amyloliquefaciens, A. calcoaceticus, B. megaterium, S. aureus,* and *B. flexus*. Gold, silver, platinum, palladium, magnetite, titanium, cadmium sulfide, selenium, titanium dioxide, and other metal nanoparticles can be produced by the previous mentioned bacteria [60] [Table 3].

**Table 3:** Biosynthesis of nanoparticles using bacteria.

**Bacteria species**	**Type of Nanoparticles**	**Size of Nanoparticles**	**Biological Activities**	**Reference**
*Lactobacillus plantarum*	Zinc oxide	7 nm	Wound healing	[61]
*Pseudomonas fluorescens*	silver	20–30 nm	Antibacterial	[62]
*Lactobacillus plantarum*	silver	4.7–24.3 nm	Antibacterial and Antioxidant Activity	[63]
*Bacillus subtilis*	Titanium dioxide	66–77 nm	Antibacterial	[64]
*Rhodopseudomonas Capsulate*	gold	5–25 nm	Bioreduction	[65]
*Lactobacillus Fermentum,*	Iron Oxide	15–20 nm	Antibacterial	[66]

### 3.10 Synthesis of nanoparticles using actinomycetes

Actinomycetes generate nanoparticles that exhibit strong biocidal activity against a range of diseases along with good disparity and stability [67], [68]. *Nocardia farcinica*, *Streptomyces viridogens*, *Streptomyces hygroscopicus* fabricated gold nanoparticles efficiently all along. Streptomyces species however, were effectively employed to produce zinc, silver, and cupper nanoparticles [69], [70].

### 3.11 Synthesis of nanoparticles using plant

Nanoparticles have been successfully synthesized from a variety of plant parts, including roots, shoots, leaves, flowers, stems, seeds, barks, and their metabolites [71], [72]. Plants with low costs and a high level of environmental friendliness are incredibly smart and beneficial to human use. Different plants extracts were used for nanoparticles production such as *Cinnamom zeylanicum, Ocimun sanctum, Anogeissus latifolia* and *Doipyros kaki* [73], [74] (Table 4). Various plant-based nanoparticles were utilized in medicine such as *Tinospora cordifolia, Juglans regia, Azadirachta indica* and *Ferula asafoetida* declared cytotoxic effect against lung cancer, breast cancer, Hematoma and colon cancer cell lines respectively. Silver nanoparticles derived from *Catharanthus roseus and Urtica dioica* possessing the ability to heal wounds in mice model. Nanoparticles derived from *Lonicera japonica* has antidiabetic potential and neuroprotective properties.

**Table 4:** Biosynthesis of nanoparticles using plant extract.

**Plant Name**	**Type of Nanoparticles**	**Size of Nanoparticles**	**Biological Activities**	**Reference**
*Desmodium triflorum.*	silver	5–20 nm	Antimicrobial	[75]
*Chrysophyllum oliviforme*	silver	20–50 nm	Antioxidant, Anticancer	[76]
*Phyllanthus amarus*	silver and gold	25–50 nm	Antibacterial	[77]
*Camellia sinensis*	Palladium	5–20 nm	Catalytic	[78]
Green tea	iron	50–80 nm	Removal of Hexavalent Chromium	[79]
*Cycas pschannae*	ZnO_2_ NPs	50–100 nm	Antibacterial	[80]
*Cyrtrandroemia nicobarica*	ZnO_2_ NPs	20–200 nm	Antioxidant	[81]

### 3.12 Biosynthesis of nanoparticles using marine algae

*Sargassum wightii*, a brown seaweed, can produce nanoparticles with a specific size range of 8–12 nm [85]. *Fucus vesiculosus*, a seaweed, capable of Au bio-absorption and bio-reduction reactions during the nanoparticle synthesis process. These reactions are beneficial for removing Au from dispersing hydrometallurgical solutions and electronic scrapes to produce NPs with a variety of sizes and shapes. Nanoparticles synthesis based on algae extracts declared a strong antibacterial activity on cotton fabrics. According to reports, *G. acerosa* highlights the importance of acquiring antifungal silver nanoparticles [85], [86].

**Table 5:** Biosynthesis of nanoparticles using aquatic algae.

**Algae Name**	**Type of Nanoparticles**	**Size of Nanoparticles**	**Biological Activities**	**Reference**
*Sargassum wightii*	gold	8–12 nm	Antibacterial	[82]
*Sargassum wightii*	silver	6–20 nm	Fabric	[83]
*Ulva fasciata*	silver	4–10 nm	Antifungal	[37]
*Cystophora moniliforms*	silver	28–35 nm	Antibacterial	[37]
*Caulerpa racemosa*	silver	5–25 nm	Antibacterial	[84]

## 4 Application of bio-nanomaterials

### 4.1 Diseases diagnosis and imaging

Diseases diagnosis is the most essential step in the healthcare improvement [87]. In order to avoid "false negative" cases, it is desirable that all diagnoses be made quickly, accurately, and specifically. Without requiring surgery, in vivo imaging is a non-invasive method that detects indications or symptoms in a patient's living tissues [88]. An earlier advancement in diagnostic imaging is able to identify cellular alterations in the tissues. A biological marker may be an early detection tool in order to identify diseases or symptoms [89]. Notably, nanotechnologies could help in the development of some of these highly precise molecular imaging agents. Imaging is essential not only for diagnosis but also for controlled drug release research, identifying potentially harmful reactions, assessing medication distribution in the body, and closely tracking the course of therapy. Potential drug toxicity can be reduced by tracking how medications are transported throughout the body and by releasing the substance as necessary [90]. Imaging methods that are well established and frequently utilized in biochemical and medical research include computing tomography, magnetic resonance imaging, ultrasound, nuclear medicine, and X-rays [91]. Nevertheless, these methods are limited in their ability to detect tissue changes early in the course of a disease. However, advancements in nanotechnology can enhance the resolution and specificity of these techniques by using contrast and targeting agents, ultimately pinpointing the site of disease within the tissue [92]. The majority of contrast agents used in medical imaging today are tiny molecules that are rapidly digested and dispersed widely, which increases the risk of harmful toxic reactions [93]. Nanotechnologies are particularly useful in the field of medicine because they improve different imaging techniques by producing more powerful contrast agents. Because of their lower toxicity and better capacity to accumulate in tissues, nanomaterials are useful in this situation and can increase the efficacy of diagnostic procedures nanoparticles size influence targeting, cellular uptake, tissue penetration, blood circulation half-life, and bio-distribution [94]. The application of nanoparticles in Xray technology is subject to certain constraints.A large number of dense atomic particles directed to the target spot without causing any negative reactions in order to improve the differentiation. The use of stable and inert surface atoms, like those present in gold, can help achieve this goal. [95]. Gold nanoshells, which contain heavy metal nanoparticles (dielectric core) enclosed in gold shells, have gained attention due to their low toxicity. They have been presented as a viable material for optical cancer imaging [95], [96]. Because they are non-invasive, gold nanoshells are inexpensive, safe, and have the potential to produce high-resolution imaging. The physical properties of gold nanoshells and gold colloids are comparable because both exhibit a uniform electronic response of the metal to light, leading to active optical absorption [97]. Researchers often use gold nanoshells as contrast agents in optical coherence tomography of cancer cells because they can precisely change their optical resonance throughout an extensive range, which includes near-infrared, whose tissue transmission is higher [98].

### 4.2 Drug delivery

**Fig. 6:**
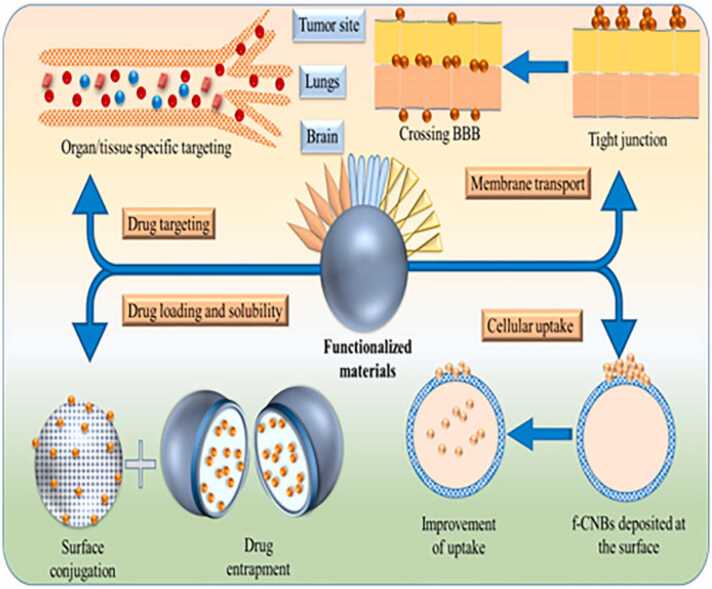
Role of nanoparticles as a drug delivery system.

Usually, therapy entails administering medication to a particular target location. External treatment approaches like radiotherapy and surgery were applied if there is no internal drug delivery channel accessible. These techniques were utilized singly or in combination to treat diseases. The aim of treatment is to eradicate the tumors or its cause [99]. With the creation of innovative drug delivery systems, nanotechnologies have contributed a significant contribution in this field. Several of these techniques are successful in clinical settings and are currently in use [100]. For instance, doxorubicin, a medication with a high level of toxicity, can target tumor cells via liposomes (Doxil®) without harming the kidneys or heart. Furthermore, paclitaxel combined with polymeric mPEG-PLA micelles (Genexol PM®) is utilized in the chemotherapeutic management of breast tumors that have metastasized [21]. Improved in vivo distribution and favorable pharmacokinetics are the main reasons for the success of nanotechnologies in drug administration [101]. The two components of an ideal medication delivery system are the capacity to target and control drug release. By precisely identifying and eliminating dangerous or malignant cells, side effects can decrease and medication efficacy can guarantee. Furthermore, controlled drug release can lessen the negative effects of medications [102].Nanoparticle drug delivery systems offer reduced irritating reactions and increased penetration, making them suitable for intravenous and other delivery methods. Nanoparticle drug delivery approaches achieve specificity by combining medications with nano-scaled radioactive antibodies that match antigens on cancer cells. This strategy has shown promising outcomes [103], improving uptake of low soluble drugs, delivery of drugs to their target sites, and drug bioavailability [104] as rep[resented in Fig. 6 [Fig. 6].

### 4.3 Biomedical applications of nanoparticles as a promising drug delivery system

#### 4.3.1 Antibacterial activity

The purpose of nanoparticles in disease-causing organisms is to disrupt the cell membrane's polymer subgroup. The challenging role of nanoparticles efficiently is damaging bacterial cell membrane and disrupting protein synthesis. Silver nanoparticles in a high concentration can rupture bacterial cell membrane and successfully destroy the bacterial cell wall. The bacterial cells exposed to silver nitrate grew more quickly than exposed to R. apiculate-mediated silver nanoparticles. This difference in growth rate could be attributed to the size of the particles and enhanced external interaction that caused induced cell membrane rupture and cell interruption [105].

Combination of both platinum nanoparticles and microorganisms to target different disease. Numerous antibiotics are effectively resistant to microbes. Various microbes exhibit resistance to different antibiotics. Meanwhile, nanoparticles have highly antimicrobial activity. Numerous investigations on metallic nanoparticles, including gold, silver, lead, platinum, zinc oxide, and titanium dioxide, have demonstrated a significant contribution to their antibacterial characteristics against pathogenic microorganisms [106], [107].

Platinum nanoparticles with significant antibacterial activity against *S. aureus* were produced biogenic using apigenin, an extract from chamomile. Furthermore, it was investigated that the growth of *E. coli* is inhibited by platinum nanoparticles [108]. Another study discovered that *Streptococcus mutans* is suppressed by a mixture of partial ammonium and platinum nanoparticles, which possessed antibacterial properties [109]. Combinations of polyamides, such as sulfones, exhibit more potent antibacterial effects against *S. aureus* and *E. coli* [110]. Silver- platinum nanoparticles loaded with particles that ranged from 2 to 3 nm demonstrated substantial antibacterial activity versus *S. choleraesuis, P. aeruginosa, K. pneumonia,* and *E. coli*. Recent research suggests that growth of bacteria was inhibited by mitochondrial membrane integrity and ATP production [111]. Furthermore, Polyvinylpyrrolidone (PVP) when coupled with platinum nanoparticles has good antibacterial activities against *Lactococcus lactis, K. pneumonia,* and *E. coli* [112]*.*

**Fig. 7:**
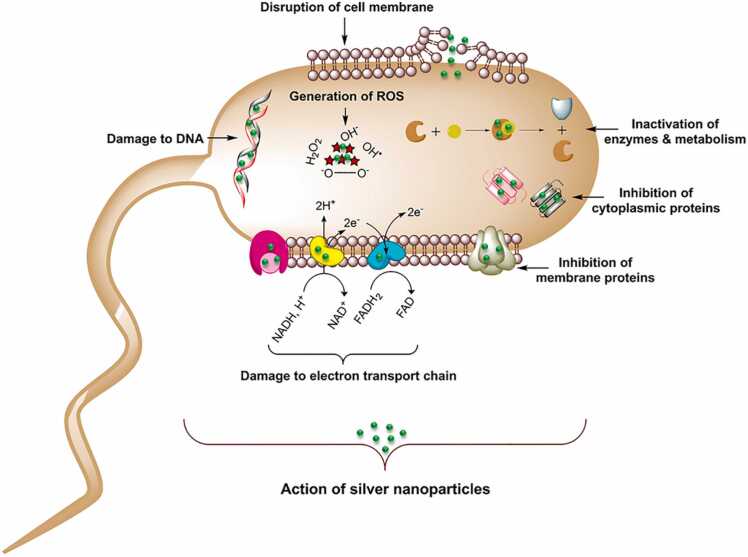
mechanism of action of antibacterial activity of biogenic silver nanoparticles [116].

Biogenic synthesis of silver nanoparticles, which derived from bacteria, fungi, algae different parts of plant or extract possess an efficient antibacterial activity as well as overcoming multidrug-resistant against numerous infectious diseases caused by different bacteria species as *S. aureus* and *S. epidermidis*. Aspergillus fungi produce powerful silver nanoparticles with antibacterial activity versus methicillin-resistant *S. epidermidis* and *S. aureus*. Silver nanoparticles synthesized with the help of *Aspergillus oryzae* filamentous mold have exhibited antibacterial effects against S. aureus KCCM 12256. Furthermore, *Bipolaris nodulosa* fungi is a potential stabilizing agent for silver nitrate, leading to the generation of silver nanoparticles in *B. subtilis* and *P. vulgaris* pathogens [113]*.* Moreover, biosynthesized silver nanoparticles were achieved using gilled mushrooms of the *Pleurotus sajor-caju* species, demonstrating effectiveness against *S. aureus* [114]*.* A previous research focused on investigating the ability of *Phoma glomerata* fungal plant pathogens to synthesize silver nanoparticles and enhance their antibacterial efficacy against *S. aureus* [115]. *Trichoderma viride,* a mold species, utilizes bio-mediation to bind with nanoparticles, showing efficacy against vancomycin-resistant *E. coli*. In comparison to alternative techniques of producing colloidal silver nanoparticles, biogenic silver nanoparticles shown superior bacterial characteristics towards *S. oneidensis*, as demonstrated by bacterial toxicity testing. The following figure represented the mechanism of action of antibacterial activity of biogenic nanoparticles [Fig. 7]. In general, smaller nanoparticle size have more antibacterial action than larger ones. This is due to their higher surface-area-to-volume ratios, which allow for more interaction with bacteria and in hence influence its antibacterial activity.

### 4.4 Fungicidal activity

**Fig. 8:**
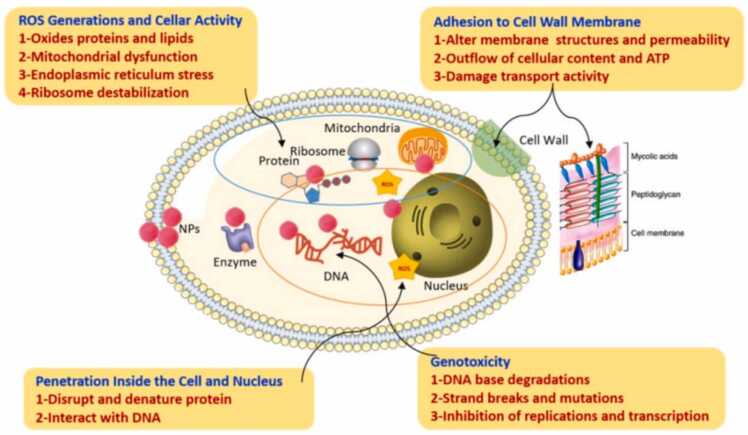
mechanism of action of Fungicidal activity of biogenic nanoparticles [120].

Alternative treatments are therefore required for fungal illnesses because general antifungal medications can have a number of side effects, including diarrhea, increased renal failure, nausea, and elevated body temperature. According to a recent study, platinum nanoparticles showed antifungal effectiveness against a number of dangerous fungi, including *P. drechsleri*, *D. bryoniae*, *C. fulvum*, *C. acutatum*, and *P. capsici*. The antifungal activity of biopolymer-based platinum nanocomposites were evaluated against a range of fungal strains. Previous studies have reported that the antifungal characteristics of platinum nanoparticles in a nano-mixture-induced membrane breakdown, increased the ROS ratio, altered the mycelia's structure, and caused cellular breakage and DNA damage [117]. Biosynthesized metallic nanoparticles have a more effective fungicidal action mechanism than widely used antibiotics like fluconazole and amphotericin. The use of silver nanoparticles derived from plant extracts has successfully exposed *Candida sp.* membrane disruption, which disrupts fungal intracellular components and causes cellular damage [118]. Certain antifungal drugs that are currently accessible have limited their application, reduced their activity, and failed to cure microbial infections. Silver nanoparticles' broad-spectrum characteristics have an intriguing effect on spore spreading of fungus and seriously hinder fungal growth. As described in the following figure, the structure of the fungal cell wall was significantly changed by exposure to nanoparticles [119] [Fig. 8].

### 4.5 Anti-plasmodial activity

The distinct management approach for the produced anti-plasmodial species is more expensive and less effective at managing the particular organism in the medical area. Nonetheless, effective and widely used antimalarial medications are still required to control plasmodial characteristics. In the past, plants were used as standard agents required for creating medications that combat malaria. Artemisinin and quinine constituents, two secondary phytochemical components, have proven to be beneficial in combating the resistant malaria parasite. In order to manage the different strains based on enhanced parasite protection, a replacement medication is urgent. Metallic nanoparticles including silver, platinum, and palladium nanoparticles, which aid in limiting the growth of malaria, were produced by the plant extract. Malarial proliferation was stopped by the bio-mediated aggregation of metallic silver nanoparticles by plant extract [121]

### 4.6 Antiviral activity

**Fig. 9:**
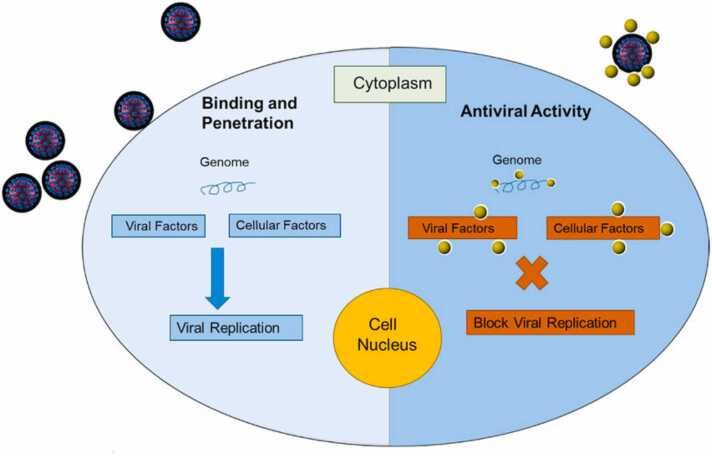
Antiviral activity of biogenic nanoparticles [128].

Target-acting medications have produced a greater 90 % treatment ratio for Hepatitis C, and antivirals have been examined in conjunction with varying degrees of completion. Due to limited availability, target-acting antiviral technology was not used to induce activity, despite the improved outcome with Hepatitis C that was discovered [122]. The pressing requirement for antiviral medication resistance has resulted in several viral infections, including those involving particular virus targets, going unfulfilled. Recent studies have discovered a connection between drugs and a particular viral target. Moreover, creating technologies that engage with modified host components may lead to the treatment of viral diseases. Host cell agents, which the virus requires for replication but which the host excretes, was one potential target. Viral proliferation is inhibited by these specificities through termination duplication, and host activity is similarly decreased [123]. The first line of defense against viral malfunction and death was the inherent viral infections. In order to trigger the immune response that resulted in tissue damage during the viral removal reaction, the secondary host was the target. Another potential target for infection cure could be the host immune response, which would limit tissue-breaking immunopathology while promoting host-mediated viral control. Antiviral therapy may also encourage cooperation with target-acting antivirals in order to host immuno-manipulation to eliminate the causes of both suffering and mortality. Plant-associated nanoparticles are used as stand-in medications for the management and treatment of viral infection [124]. The virus poses a serious threat to the host when it enters, and it needs to adapt more quickly in order to grow. Silver nanoparticle biosynthesis may be an effective means of producing broad-spectrum antiviral agents that restrict the characteristics of virus cells. The bio-mediated produced silver nanoparticles have potent anti-HIV agents during the first stage of the reverse transcription mechanism [125]. Effective antiviral compounds found in nanomaterials inhibit the virus before it can infect the host system. The metallic nanoparticles that are bio-mediated generated exhibit diverse coupling actions that allow them to engage with viral cell populations and regulate their characteristics. The bio-associated nanoparticles have a potent, all-around antiviral effect on both cell-free and cell-mediated viruses. Moreover, gold and silver nanoparticles significantly inhibit the HIV-1 life cycle prior to infection. Moreover, metallic nanoparticles have the ability to neutralize retroviruses [125]. Magnesium nanoparticles, particularly stabilized ones, have a higher number of interactions with host cells and viruses compared to other types of nanoparticles. Metallic nanoparticles antivirus can occur both within and outside the host cell. The process inhibited virus adherence to host cells and ended host cell coupling positions when nanoparticles interacted with gp120 proteins. Dispersing the virus fragments prior to their entry into the cell led to viral genome coupling, which is another plausible method. Applying bio-nanoparticles as antivirus agents would involve attaching the materials to the virus's surface [126]. Whereas silver nanoparticles inhibit viral proliferation, coupling, and arrival, gold nanoparticles stop the gp120 coupling to CD4 and inhibit the virus's entry. By making a connection with the viral gp120 in cell-mediated viruses, silver nanoparticles stop CD4-associated virion coupling, amalgamation, and pathogenicity. Silver nanoparticles (NPs) in dual-standard viruses stop the virus from growing after they make contact with the viral DNA. Viral DNA polymerase activity is disrupted by zinc nanoparticles, which stops the virus from growing. Furthermore, the size of zinc oxide nanoparticles may prevent virus entrance into the cell [127] [Fig. 9].

### 4.7 Anti-inflammatory activity

Anti-inflammatory therapy works by inducing immunological reactive compounds such interleukins and cytokinins, which can lead to the development of keratinocytes, T, B, and C lymphocytes, and macrophages. Numerous anti-inflammatory mediators, including enzymes and antibiotics, are produced by the endocrine system. The key immune organs create cytokines (IL-1 and IL-2), another important anti-inflammatory molecule. These anti-inflammatory substances promote the healing process. Inflammatory arbitrators regulate the spread of disease via occurring in biochemical pathways. Silver nanoparticles that are obtained through bio-mediated means improve the wound healing process and promote tissue regrowth in inflammatory characteristics. One study suggested that bio-mediated gold and platinum nanoparticles are effective alternatives to conventionally prescribed medications for inflammation treatment [129].

**Fig. 10:**
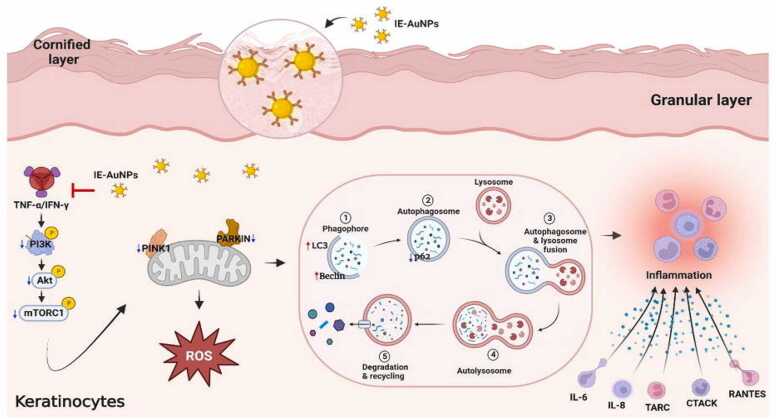
Anti-inflammatory activity of biogenic nanoparticles [132].

Nanoparticles have been explored as anti-inflammatory agents in recent years. High exterior-position-to-interior ratio nanoparticles are utilized to block other supplements and inflammatory substances such cytokines and inflammation-mediating enzymes. There have been reports of several metal-mediated nanoparticles with strong anti-inflammatory qualities, including those based on gold, silver, iron oxide and copper [37]. The body's initial reaction to internal disruption, transmission, hormone imbalances, and harm to internal structure and exterior functions such as infection by pathogenic microorganisms or an external element is swelling. Antigen receptors on an individual basis initiate metabolic reactions. Damage to tissues and cells influences inflammation, resulting in differences in the signals controlling the inflammation. Tissue that has been damaged or infected generates an inflammatory response that leads to the development of killer cells and macrophages. Auto-inflammatory reactions are controlled by macrophages. Large, single-nucleated phagocytes known as macrophages originate in the bone marrow when mature white blood cells move from the circulation to become monocytes. After that, these monocytes migrate to different tissues where they mature into macrophages. Pro-inflammatory M1 macrophages, whose progression causes inflammatory disorders, and anti-inflammatory M2 macrophages, which are stimulated as an anti-inflammatory response and cause the reassembling of the injured tissues and organs, are the two stages of macrophage evolution. Macrophages can tolerate the inflammatory response by inducing the two traits that are unexpected in retarder's illness. Through phagocytosis, the macrophages eliminate the inflammation and skin damage, which causes inflammation through initiation signals that trigger the macrophages [130], [131] [Fig. 10].

### 4.8 Antidiabetic activity

**Fig. 11:**
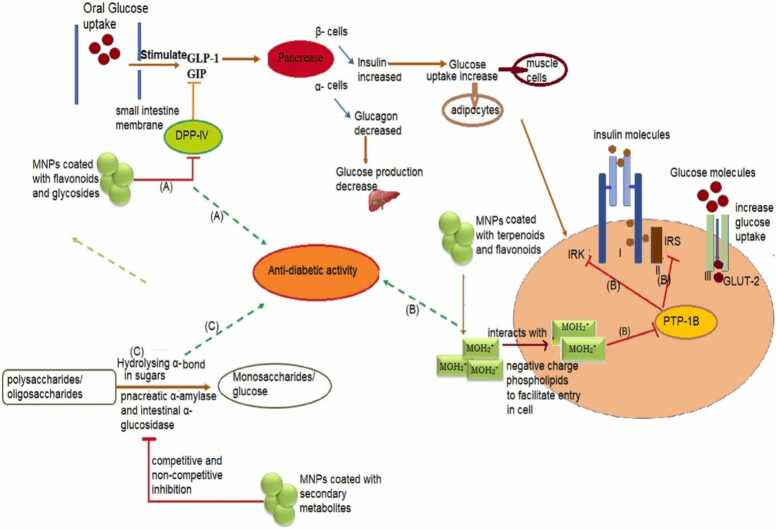
Anti-diabetic activity of biogenic nanoparticles [136].

In certain food scales, certain foods, stability diets, and synthetic medications may suppress diabetes, making DM management challenging. Moreover, the biosynthesized nanoparticles could treat diabetes mellitus as a replacement medication [Fig. 11]. Daisy and Saipriya (2012) claim that gold nanoparticles have superior therapeutic activity for managing diabetes. In diabetic mice, the exposure to gold nanoparticles successfully reduced the ratio of liver enzymes including alanine movement, serum creatinine, uric acid alkaline, and phosphatase. Furthermore, when gold nanoparticles were exposed to a diabetic animal, the glycated hemoglobin scale that was controlling the standard scale decreased [133]. Biogenic produced silver nanoparticles from *Sphaeranthus amaranthoides* was previously investigated reduced a-amylase and carbohydrate levels [37]. Extract from *Premna herbacea Roxb* has the ability to prevent diabetes [134]. Furthermore, it was reported that, nanoparticles are crucial therapeutic agents for improving diabetes control. The sugar ratio of 140 mg/dL in upon silver nanoparticles treatment was fully controlled in the medicinal research conducted in mice [135].

### 4.9 Antioxidant activity

Enzymes and non-enzymatic agents in addition to antioxidant activity control free radical generation. In addition to brain damage, malignancy, and heart disease, radicals can damage the cells. Free radicals are produced by ROS, including SOD and hydrogen peroxide. Bio-constituents that efficiently control the production of free radicals include proteins, lipids, glycoproteins, phenolic, and flavonoids. Additionally, the ability of antioxidants to scavenge pathogens is crucial for managing a number of illnesses, including metabolic and neurological conditions. When it comes to antioxidants, silver nanoparticles outperform conventional medications like ascorbic acid. Both the tea extract and the nanoparticles demonstrated higher levels of flavonoids and phenolic components as well as improved antioxidant activity. Free radicals and reactive oxygen species have shown biological system activity, according to Yazdi et al. [127]. These agents are byproducts of normal metabolism that harm cell development, causing cell rupture, cellular constituent inaccuracy, and destruction to shared characteristics across multiple cells. Oxidative stress contributes to the spread of diseases such as cancer, Alzheimer's, and blindness. Plants contain many antioxidants, which protect human health because they strongly preserve biological systems against these substances, select toxic free radicals, and lower cell destruction. The nanomaterial is familiar as a vehicle system for selected drug transmission in current years [128].

**Fig. 12:**
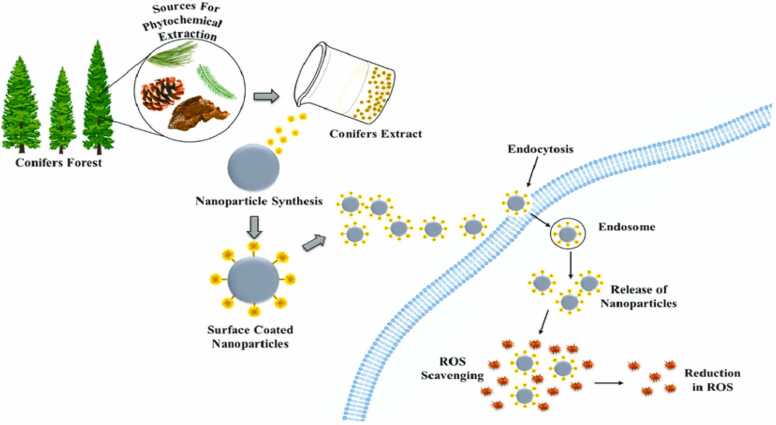
Antioxidant activity of biogenic nanoparticles [137].

Ceric oxide nanoparticles utilized as cancer therapy vehicles may normalize ROS because to their antioxidant properties. According to the above-mentioned studies, these nanoparticles exhibit anti-cancer properties while also acting as antioxidants and protecting healthy cells [129] [Fig. 12].

### 4.10 Anticancer therapy

A staggering number of people globally are suffering from cancer, highlighting the necessity for a highly accurate technique of detection and a unique drug delivery system that is more efficient, targeted, and has fewer adverse effects [138]. Anticancer therapies are frequently seen to be better if the therapeutic drug can reach the intended target site without producing any negative side effects.

Gold nanoparticles have particular benefits for drug delivery and medical diagnostics, making them a popular choice in numerous biomedical applications. Their remarkable biocompatibility makes them ideal for biomedical applications that need little cytotoxicity. Gold nanoparticles are more stable and less toxic due to gold's chemical inertness. Whereas, silver nanoparticles, for instance, are more toxic and can have negative health impacts as compared to gold nanoparticles [138].

The application of various nanoparticles has allowed for several improvements in recent years. In this section, several of the nanoparticles are featured. In the treatment of HT-29 cells in colon cancer, silver nanoparticles derived from the probiotic bacterium *L. rhamnosus* GG were particularly beneficial [139]. Excellent antitumor activity was demonstrated by gold nanoparticles from *L. kimchicus* DCY51T against human lung adenocarcinoma cell line A549 and human colorectal adenocarcinoma cell line HT29. *L. casei's* selenium nanoparticles were incredibly effective against colon cancer cells [139]. Gold nanoparticles derived from *L. casei* were shown to be efficacious against human colon cancer cell line (HT-29) and human gastric cancer cells (AGS) [140]. It was discovered that platinum nanoparticles derived from *Streptomyces sp.* were efficient against the MCF-7 cell line, a kind of breast cancer [141]. Moreover, it was discovered that selenium nanoparticles derived from Lactobacillus species was efficacious against human colon cancer (HT-29) [140]. It follows that many nanoparticles derived from the biosynthetic technique have demonstrated efficacious anticancer properties.

**Fig. 13:**
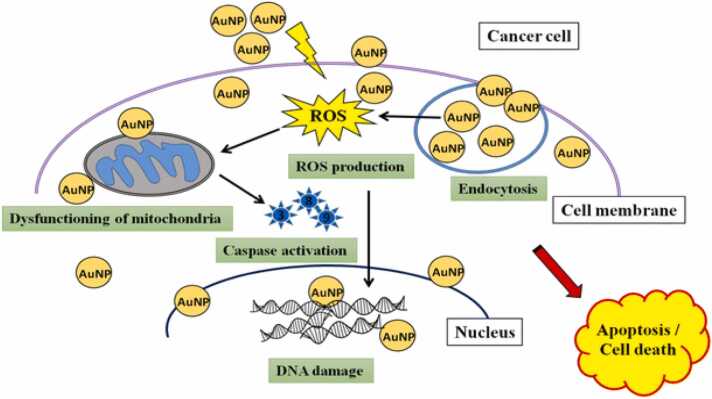
Anticancer activity of biogenic nanoparticles [146].

Chemical surface changes on nanoparticle carriers could enhance the necessary targeted delivery. Polyethylene oxide, or PEG, inclusion is one of the best examples of surface changes at the nanoparticle level [142]. These alterations improve the drug's capacity to target tumors as well as its specificity of absorption. By using PEG, nanoparticles can travel through the circulation until they reach the tumor because the body's immune system won't recognize them as foreign items. Hydrogel's use in the treatment of breast cancer is another excellent example of this cutting-edge technology. Herceptin is a class of monoclonal antibody that targets human epidermal growth factor receptor-2 in breast cancer treatment. This has led to the development of a vitamin E-based hydrogel that, with a single dosage, can carry Herceptin to the target region for several weeks. The hydrogel-based drug administration is more effective than traditional subcutaneous and intravenous delivery methods because Herceptin is better retained within the tumor, which makes it an effective anti-tumor agent [143]. Through the application of nanotechnologies, nanoparticles can be altered in a variety of ways to improve drug localization, extend circulation, boost efficaciousness, and possibly prevent the emergence of multidrug resistance [144]. Numerous studies have used FDA-approved nanoparticle medications as adjuvants in combination cancer treatments, such as Abraxane®, Doxil®, or Genexol-PM®. Metastatic breast cancer patients can now receive treatment with Abraxane®, a paclitaxel albumin-stabilized nanoparticle formulation (nab-paclitaxel) [145]. Among the anticancer drugs in liposome-based drug formulations that have been studied the most are vincristine, paclitaxel, doxorubicin, and daunorubicin. The liposomal lipid layers have both hydrophilic and hydrophobic terminals, which allows for the simultaneously delivery of polar, amphiphilic, and hydrophobic drugs which can be achieved as an efficient anticancer drug delivery system. Mechanism of action of anticancer activity of biogenic nanoparticles is represented in Fig. 13 [Fig. 13].

### 4.11 Bio-sensing applications

**Fig. 14:**
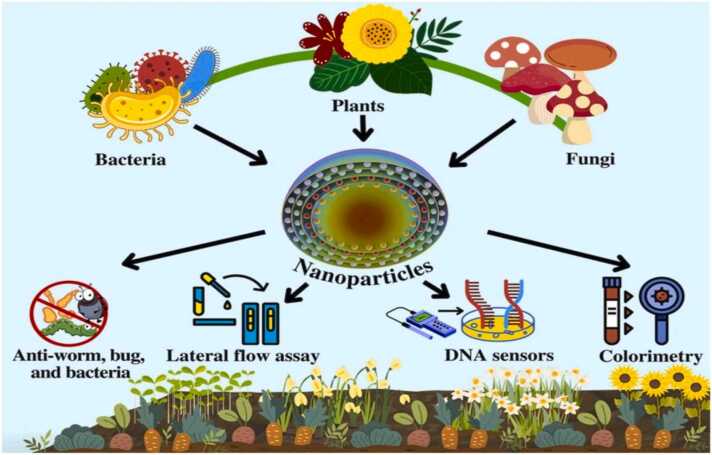
Bio-Sensing applications of biogenic nanoparticles [153].

**Fig. 15:**
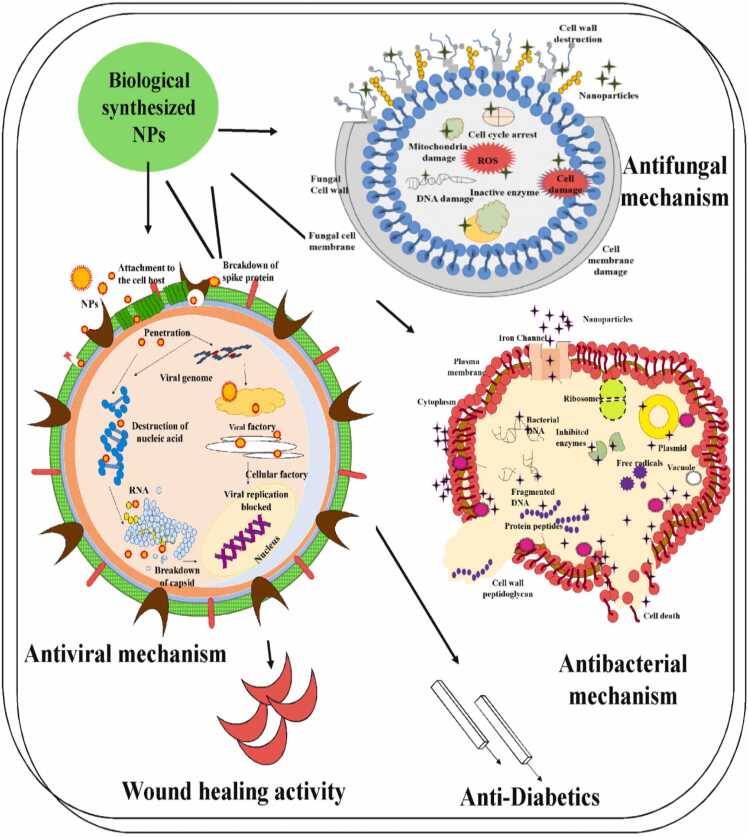
Benefits of biogenic nanoparticles in disease treatment [37].

As represented in Fig. 14 [Fig. 14], the employment of nanoparticles in biological material sensing is highly beneficial [147]. These kinds of bio-sensing applications involve the utilization of many nanoparticles [148]. In one work, cancer was examined via the green synthesis of gold-silver nanoparticles mediated by chloroplasts [149]. Platinum nanoparticles produced by *S. myriocystum* were employed to identify asthma and allergies [150]. The synthesis of gold nanoparticles mediated by *Hypnea valencia* was utilized to identify pregnancy in females [151]. Moreover, the ability of *Noctiluca scintillan*s-mediated silver nanoparticles production to identify problems with mouth gums and oral discharge was assessed [152]. The following figure illustrated the role of biogenic nanoparticles in medicine suggesting that it could be a promising technology in bio-health and as a novel drug delivery system [Fig. 15].

## 5 Conclusions and future scope

The discipline of nanotechnology generates novel biomedical nanoparticles for use in pharmaceutical and therapeutic applications, and it is primarily tied to the fields of physics, chemistry, biology, and material science. The substantial applications of nanoscience in the chemical, pharmaceutical, medical diagnosis, electronics, space, agriculture, and illness healing industries have attracted the attention of researchers in recent years. Nowadays, it's believed that nanoparticles are incredibly beneficial materials. Actinomycetes, bacteria, fungus, plants, and yeast are some of the organisms that provide the biologically mediated nanoparticles. With less harmful side effects, bio-mediated nanoparticles have been extensively employed to treat a wide range of pathogenic disorders. Furthermore, metallic nanoparticles mediated by biological processes are less costly, less hazardous to the environment, and non-toxic. Moreover, employing genetic engineering techniques, the produced nanoparticles may increase activity. Certain characteristics of bio-mediated nanoparticles include increased biocompatibility, increased surface area, increased reactivity, and lack of toxicity. A clear perspective on the several sources of synthetic nanoparticles and their medical uses, including antibacterial, antifungal, antiviral, anti-inflammatory, antidiabetic, and antioxidant properties.

A thorough analysis of the risks associated with nanomaterials and their use in biological systems is still lacking. This necessitates the development of a uniform technique for measuring toxicity or other harmful impacts on people and the environment. The public's acceptance of nanobiotechnology, safety concerns, risk assessment, and regulation are a few of the major obstacles it may encounter. To achieve a successful application, understanding the structure-function relationship of nanomaterials is crucial. Finding the ideal nanomaterial for a given use will be difficult, but so will producing nanomaterials in an economical, scalable, and sustainable manner. Biodegradable nanoparticles could be used to deliver treatments that are either water soluble or insoluble and have better biocompatibility and retention duration. The industrial sector has the potential to extend the scope of nanobiotechnology by producing substances such as the nanoadditives and nanozymes. Medical equipment and technologies can be designed to create highly selective molecular or subcellular interactions.

## 6 Role of Egyptian research in biogenic nanotechnology

In Egypt, many research groups have concerned using biological systems for the synthesis of nanoparticles due to the numerous benefits they offer over non-biological systems. A number of factors contribute to the unique qualities and applications of nanoparticles, including their size resemblance to biomolecules like polynucleic acids and proteins. The biogenic technique produced nanoparticles with good stability, dimensions, and variability. The biogenic technique produced nanoparticles with good stability, dimensions, and polydispersity. Methods including chemistry, biology, and physical synthesis are used to create the nanoparticles. The chemical and physical techniques are quite expensive. Because biological techniques of synthesizing nanoparticles enable synthesis to occur at physiological pH, temperature, and pressure while also requiring very little money, they can help eliminate harsh processing conditions.

According to Abdel-Fattah et al., two plant extracts were used to environmentally manufacture biogenic Ag@Pd core-shell nanoparticles with the goal of enhancing their anticancer and antibacterial properties. The produced biogenic core-shell nanoparticles are suggested for bactericidal and cancer therapeutic applications with enhanced efficacy. Individual structures can be tuned to yield tailored qualities [154].

Salem et al. synthesized iron oxide nanoparticles using *Colpomenia sinuosa* and *Pterocladia capillacea* seaweed aqueous extracts. The results exhibited broad range of antibacterial effectiveness against the development of +ve and -ve Gram bacteria. Additionally, it demonstrated outstanding antifungal efficacy against *Fusarium oxysporum* and *Aspergillus flavus* [155].

Abdelaziz et al. isolated two fungal strains from local Egyptian soil samples to biosynthesize silver and gold nanoparticles. Furthermore, Abdel-Moneim et al. evaluated the antibacterial and antioxidant properties of three *Spirulina extracts* and the biological selenium nanoparticles manufactured by *Bacillus subtilis* AL43 [156].

Aboelmaati et al. synthesized biocompatible and biogenic silver nanoparticles by the agro-waste *Hibiscus sabdariffa* stem. They demonstrated a six-fold increase in IC50 value over the control cell line in their apoptotic anti-ovarian cancer action. The polydopamine-functionalized silver nanoparticles, when coupled to either *moxifloxacin* or *gatifloxacin*, demonstrated enhanced antibiofilm activity against biofilms of *Klebsiella pneumoniae*, Pseudomonas aeruginosa, and *Acinetobacter baumannii*. The level of activity was considerably higher than that of the antibiotics either alone or together with enriched silver nanoparticles, indicating a synergistic effect.

Moreover, Eid et al. synthesized bacterial eco-friendly silver nanoparticles using *Bacillus amyloliquefaciens* Fa.2. Ag-NPs produced by bacteria shown multifunctional properties that could be applied in biomedical and environmental catalysis [157].

In cancer research, several investigation concerned with the development of anticancer drug delivery system. For instance, El Deeb et al. evaluated a green manufacture of novel stable biogenic gold nanoparticles for the induction of intrinsic and extrinsic pathways as treatments for breast cancer [158].

Nowadays, our teamwork developed a novel inorganic nanoparticles biosynthesized at National Research Centre labs.

## CRediT authorship contribution statement

**Rehab M. Abdel-Megeed:** Writing – review & editing, Writing – original draft, Project administration, Data curation, Conceptualization.

## Declaration of Competing Interest

The authors declare that they have no known competing financial interests or personal relationships that could have appeared to influence the work reported in this paper.

## Data Availability

No data was used for the research described in the article.
